# Openness to Experience as a Predictor and Outcome of Upward Job Changes into Managerial and Professional Positions

**DOI:** 10.1371/journal.pone.0131115

**Published:** 2015-06-25

**Authors:** Christiane Nieß, Hannes Zacher

**Affiliations:** 1 Cologne Graduate School in Management, Economics, and Social Sciences, University of Cologne, Albertus-Magnus-Platz, Köln, Germany; 2 Department of Psychology, University of Groningen, Grote Kruisstraat 2/1, Groningen, The Netherlands; Institutes for Behavior Resources and Johns Hopkins University School of Medicine, UNITED STATES

## Abstract

In industrial and organizational psychology, there is a long tradition of studying personality as an antecedent of work outcomes. Recently, however, scholars have suggested that personality characteristics may not only predict, but also change due to certain work experiences, a notion that is depicted in the dynamic developmental model (DDM) of personality and work. Upward job changes are an important part of employees’ careers and career success in particular, and we argue that these career transitions can shape personality over time. In this study, we investigate the Big Five personality characteristics as both predictors and outcomes of upward job changes into managerial and professional positions. We tested our hypotheses by applying event history analyses and propensity score matching to a longitudinal dataset collected over five years from employees in Australia. Results indicated that participants’ openness to experience not only predicted, but that changes in openness to experience also followed from upward job changes into managerial and professional positions. Our findings thus provide support for a dynamic perspective on personality characteristics in the context of work and careers.

## Introduction

Personality characteristics, and the Big Five in particular, have been studied extensively as predictors of work outcomes over the past decades [1]. They predict a broad variety of organizational phenomena, including career mobility [2], career success [3], leadership [4], and job satisfaction [5]. Given that upward job changes into managerial and professional positions are related to all of these organizational phenomena, surprisingly few studies have investigated whether such upward job changes may likewise have dispositional causes. Upward job changes are widely considered to be an important indicator of extrinsic career success [6]. Since indicators of extrinsic career success are generally viewed positively by the individuals involved, other people, and society more broadly, identifying dispositional causes for such job changes is of great importance. Judge and colleagues [7] have pointed out that extrinsic career success is strongly positively related to occupational status, which is an important dimension in social interactions. Moreover, upward job changes are an objective accomplishment that is visible to third parties and are likely to be regarded positively in society. The first aim of the present study is therefore to contribute to an emerging area in the career literature [8, 9, 10, 11, 12] by investigating the Big Five as possible antecedents of subsequent upward job changes into managerial and professional positions.

Conceptualizing personality characteristics as potential predictors of organizational phenomena, such as upward job changes into managerial and professional positions, is inherent in most of the literature on the role of personality in the work and career context (i.e. [1], [2], [3], [4], [5]). It relies on the assumption that personality is temporally stable and must therefore predict work outcomes and not vice versa [13]. However, already in the 1980s, Kohn and Schooler [14] suggested that certain aspects of one’s job (e.g., work complexity) may influence personality development, and Frese [15] discussed the importance of occupational socialization for psychological development.

This notion has recently been revisited by scholars in the field of personality psychology, who developed the *dynamic developmental model (DDM)* of personality and work [16]. While the researchers who developed this model acknowledged the importance of studying personality characteristics as predictors of work outcomes, they also pointed out that this approach has resulted in two limitations in this area of research. First, Woods and colleagues [16] argued that by investigating the effects of personality characteristics on work outcomes in cross-sectional research or longitudinal studies relying on two measurement points, the relationships between personality and outcomes is treated as static. Such research designs do not account for the possibility that the relationship may dynamically change over time. Second, the researchers criticized that in the vast majority of studies in the field of organizational behavior, personality characteristics are solely treated as predictor variables. However, since work is a core part of most people’s lives today, it can be argued that certain work experiences, such as changing work and life roles, could have an influence on personality development as well. They therefore suggested that personality should also be investigated as a dependent variable, “with the focus on the reciprocal influences between personality and work” (p. 8). Consequently, the DDM states that personality characteristics may not only serve as predictors of work and career experiences, but that work and career experiences may also lead to changes in personality characteristics over time [11, 16].

Only very few studies so far have explicitly investigated reciprocal influences between personality and work (for an overview of those studies, see [16]). Therefore, little is known about which specific work experiences have the potential of evoking changes in employees’ personality characteristics. Based on the DDM of personality and work, the second aim of our study is to investigate whether upward job changes into managerial and professional positions lead to changes in the Big Five over time. We base our analyses on a large longitudinal dataset which, in comparison to cross-sectional studies, allows us to investigate the reciprocal influences between personality and job changes over time. Overall, we intend to contribute to the literatures on careers and personality by examining reciprocal effects between personality characteristics and upward job changes into managerial and professional positions by applying two advanced statistical techniques (event history analyses and propensity score matching) to this large longitudinal dataset.

### Definitions of the Big Five and Upward Job Changes

The Five-Factor Model is the predominant theoretical framework to investigate associations between personality characteristics and work outcomes (e.g. [1]). The *Big Five* include [17, 18]: openness to experience (being imaginative, independent-minded, and autonomous), extraversion (being assertive, energetic, and sociable), conscientiousness (being responsible, dependable, and orderly), agreeableness (being cooperative, trusting, and caring), and emotional stability (being calm, secure, and resilient). Importantly, in the personality literature, the construct of emotional stability is also often referred to as neuroticism, the reverse of emotional stability. In industrial and organizational psychology, meta-analyses have shown that some of the Big Five characteristics are related to, for instance, leadership behavior [4], job performance [19], and job satisfaction [5].

Career researchers defined job changes to entail “substantial changes in work responsibilities, hierarchical levels, or titles” [20, p. 352], and we argue that upward job changes into managerial and professional positions include all of these three aspects of job changes. First, employees who enter managerial and professional positions are required to make use of a different skill set, take part in specialized trainings, or take on leadership roles [21, 22]. They thus experience a substantial shift in work responsibilities. Second, managers and professionals operate on a higher organizational level than technicians, tradesmen, workers, or laborers, so that career transitions into such positions are accompanied by promotions into higher hierarchical levels [23]. Third, job titles in managerial and professional positions, such as manager, chief psychologist, or owner of a construction company clearly differ from job titles in non-managerial and non-professional positions, such as clerk, psychological assistant, or construction worker [24, 25]. In sum, moving into managerial and professional positions involves substantial changes in employees’ job responsibilities and their work environment.

### Effects of the Big Five on Upward Job Changes into Managerial and Professional Positions

According to several prominent theories in the career literature, such as the theory of vocational choice [26], person-environment fit theory [27, 28], and the attraction-selection-attrition model [29], personality characteristics may serve as predictors of people’s career-related decisions. The main conclusion of these theories is that individuals self-select into work environments that provide a good fit with their personality, a notion that has received substantial empirical support [30]. In the present study, we aim to investigate whether individuals’ upward job changes into managerial and professional positions can likewise be explained on the basis of their dispositions. This question is particularly important against the backdrop that job changes have become a salient attribute of today’s careers [31] and upward job changes into managerial and professional positions in particular constitute a form of career success. Upward job changes into these positions may also be important for individuals because through gaining new and diverse work experiences and skills in such positions, employability can be enhanced. Previous research has shown that employees differ in their attitudes toward job mobility and in the way they perceive mobility opportunities [20], and only very few empirical studies have so far examined relationships between the Big Five and actual job changes across time [8, 9, 10, 11, 12]. Those studies, however, either relied on cross-sectional data or did not focus on upward job changes into managerial and professional positions, which are particularly relevant for employees’ career success [32].

The present study aims to contribute to this area of research by investigating associations between the Big Five personality characteristics and upward job changes into managerial and professional positions. Based on conceptualizations of the Big Five characteristics, we are able to establish their effects on upward job changes into managerial and professional positions. According to a review by Feldman and Ng [20], openness to experience and extraversion are likely to be the personality characteristics that are especially important in explaining upward career mobility. The authors argue that “individuals with these traits tend to be more active and skillful in seeking out new job opportunities” ([20], p. 362). We argue that seeking out new job opportunities is a conceptually closely related constructs to and an important precondition for upward career mobility, and thus we propose effects of openness to experience and extraversion on upward job changes. Therefore, we develop specific hypotheses for the effects of those two characteristics in explaining upward job changes into managerial and professional positions. For the other three personality characteristics in the Big Five framework, namely conscientiousness, agreeableness, and emotional stability, we do not offer specific hypotheses, but describe why we do not expect them to affect upward job changes into managerial and professional positions. It is important to note, however, that we included all of the Big Five personality characteristics in our analyses testing whether the Big Five serve as predictors of job changes into managerial and professional positions.

#### Openness to Experience

Individuals with high openness to experience are curious and have a wide array of interests [33], which predisposes them to desire new experiences by moving into different jobs and positions. They also have a strong need for change and novelty [33], are prone to “job hopping” ([6], p. 625), and have been found to display a greater job instability than others [10]. Individuals with high openness to experience can further be characterized by their intellectual abilities and flexibility [6], which may lead them to seek intellectual stimulation in their occupation by taking on more challenging jobs on higher hierarchical levels. Openness to experience is also strongly related to divergent thinking [34] and creativity [35], and one of its facets is the generation of new ideas [33]. Those characteristics are in turn linked to leadership in organizations [4, 36], so that employees with high openness to experience may be especially fitting for managerial positions. Additionally, employees with high openness to experience are more likely to seek work in complex, self-directed positions [37] and jobs with higher job status [6], such as managerial and professional positions.

*Hypothesis 1*: Openness to experience positively predicts upward job changes into managerial and professional positions.

#### Extraversion

Several of the facets of extraversion, such as ambition, assertiveness, activity, and excitement-seeking [33], suggest that high scores on this personality characteristic predispose employees to seek out new challenges in their careers. Due to those dispositions, extraverted individuals should be more likely to actively deal with unsatisfactory job experiences by initiating changes [3]. Extraverts indeed switch organizations more frequently than others [10] and pursue employment alternatives by initiating job search behaviors [38]. Extraversion has furthermore emerged as one of the main predictors of job performance, especially in occupations that involve social interaction [19]. Extraverts tend to be energetic and socially dominant, characteristics that are generally perceived as relevant for leadership positions [39].

Since extraverted employees should have both the ambition and the skills to take on jobs at higher hierarchical levels, they may be especially likely to experience upward job changes into managerial and professional positions. This may be due to the fact that organizational decision makers are likely to regard extraverted employees as well-suited for positions that require frequent social interactions and leadership behaviors (e.g. managerial positions; [4, 7]). This notion is supported by empirical findings suggesting that extraversion is the Big Five trait that is the strongest correlate of both leader emergence and leadership effectiveness [4]. Overall, previous research supports the notion that extraversion predicts job changes up the organizational hierarchy, showing that extraversion has been linked to several indicators of extrinsic career success, including occupational status [40], job level [41], managerial advancement [42], and promotions [3]. However, most of this work was cross-sectional and thus did not allow the investigation of effects of extraversion on subsequent upward job changes over a period of time.

*Hypothesis 2*: Extraversion positively predicts upward job changes into managerial and professional positions.

#### Conscientiousness

Conscientiousness is the Big Five characteristic that has been shown to most consistently predict a variety of job performance criteria across a number of occupational groups [19]. Several facets of conscientiousness, such as competence, achievement-striving, self-discipline, and deliberation suggest that it should be related to career success [7]. Employees with a strong achievement orientation have indeed been found to experience greater upward career mobility [43] and managerial advancement [44]. According to Judge and colleagues [6], high conscientiousness enables employees to obtain promotions into jobs with a higher complexity and prestige. Therefore, one could argue that conscientious employees may be prone to experience upward job changes into managerial and professional positions. However, Ng and colleagues [45] have pointed out that since conscientiousness is also associated with high levels of dutifulness and deliberation, conscientious employees may prefer to stay in the same job and organization due to their high dependability and sense of responsibility. Meta-analytic findings indeed suggest that conscientiousness negatively predicts turnover decisions [46], and research has identified conscientiousness as one of the main predictors of job satisfaction, suggesting that conscientious employees may be more likely to remain in their current occupation rather than pursuing alternative options [6]. Another facet of conscientiousness, namely risk aversion or cautiousness, further supports the notion that conscientious employees may be less likely to seek out novel job opportunities, and especially managerial and professional positions. Thus, overall, we do not offer a directional hypothesis on the role of conscientiousness in predicting upward job changes into managerial and professional positions. This assessment is in line with the empirical finding that conscientiousness was unrelated to extrinsic career success in a sample of executives from the United States and Europe [47].

#### Agreeableness

For agreeableness, we also argue that it may either positively or negatively predict upward job changes into managerial and professional positions. On the one hand, agreeable employees are compliant and altruistic, and they typically get along well with others [33]. They may therefore be regarded as especially well-suited for leadership positions in which cooperation and teamwork are required [48], and thus experience upward job changes especially into managerial and professional positions. On the other hand, agreeableness is also associated with a need for affiliation [49] and agreeable employees are typically not very competitive or demanding [33]. They value getting along with others more than pursuing their self-interests [10] and may be too soft-hearted and trusting to get ahead in their careers [3]. Therefore, agreeable employees may be prone to remain in the same job [43] or even sacrifice their own career success for the sake of pleasing others [6].

#### Emotional Stability

Emotional stability is associated with good emotional adjustment and high levels of self-esteem, both of which are especially important in higher status occupations [6], and are linked to leadership effectiveness [4]. Due to their high levels of self-confidence, emotionally stable employees may be more likely to apply for new jobs, and for promotions into managerial and professional positions in particular. Individuals who score high on emotional stability furthermore typically demonstrate low nervousness and low social anxiety, so that they may be likely to seek out upward job changes. It could thus be argued that emotional stability positively predicts upward job changes into managerial and professional positions. However, emotional stability is also the characteristic that most consistently predicts job satisfaction [5], so that employees may be less likely to be willing to leave their current position. This notion is supported by the meta-analytic finding that emotional stability is negatively related to turnover intentions [43] and voluntary turnover [46]. Also, Feldman and Ng [20] have pointed out that neuroticism, which is the inverse of emotional stability, is a particularly important predictor of general job mobility. More specifically, employees high in neuroticism are likely to change jobs because they “have low self-esteem and tend to search for positive affirmation elsewhere” (p. 362). In reverse, this would suggest that employees high in emotional stability are more likely to remain in their current job and organization. In sum, due to these potentially countervailing effects, we do not offer a hypothesis on the role of emotional stability in explaining upward job changes into managerial and professional positions.

### Effects of Upward Job Changes into Managerial and Professional Positions on Changes in the Big Five

Over the past decade, empirical evidence has emerged in personality and lifespan psychology suggesting that personality changes across the adult lifespan [50] and in response to major life events [51]. A few studies in organizational psychology have shown that work experiences may likewise shape personality over the working lifespan. First, Kohn and Schooler [37] found that employees who worked in complex jobs became more intellectually flexible within the timeframe of 10 years. Second, Roberts, Caspi, and Moffitt [52] found that several aspects of employees’ work experiences, such as occupational attainment, job satisfaction, and job involvement served as predictors of changes in personality, which were assessed via the Multidimensional Personality Questionnaire [53]. Third, Jackson and colleagues [54] showed that lower levels of agreeableness, neuroticism, and openness to experience did not only predict self-selection into the military after high school, but that those participants who had entered military service reported lower levels of agreeableness five years after their service in comparison to a control group. Fourth, a recent study by Wille and De Fruyt [11] showed that the Big Five personality characteristics shape and are shaped by occupational characteristics [26] over a time span of 15 years. Fifth, another recent study came to the conclusion that work characteristics and proactive personality influence each other reciprocally [55].

The effects of career changes on personality characteristics has only recently received empirical attention, so empirical evidence is still very limited in this area. However, we base our argument on a well-developed theory, the dynamic developmental model (DDM) of personality and work [16]. The model suggests that personality should not only be regarded as an independent variable fostering certain work experiences, but that personality characteristics may also serve as dependent variables of career-related events. Building on Roberts and colleagues’ [52] theory and findings, the DDM also entails the corresponsive principle, which suggests that the personality traits that predict certain work experiences are the same ones that change in response to those experiences. In other words, the DDM proposes that personality characteristics and work experiences influence each other reciprocally and in accordance with the corresponsive principle. The changes in personality in response to work experiences can be explained in more detail by at least two theoretical frameworks, namely *trait activation theory* [56] and *social investment theory* [57].

Trait activation theory suggests that personality characteristics require relevant situations to be expressed, which is referred to as the trait-activation potential of the situation [56]. Applied to the context of the present study, upward job changes into managerial and professional positions may provide employees with new situations that have a different trait-activation potential than previous situations. Employees are then required to make use of the appropriate personality characteristics when they are confronted with those novel situations. By consistently behaving according to the requirements of the situation, those characteristics may then be enhanced. For example, employees may change into positions that involve showing leadership behaviors. In those new positions, they are likely to be confronted with situations that have a stronger trait-activation potential for openness to experience and extraversion than their previous positions did. They are therefore required to behave in a more open and extraverted way and subsequently perceive themselves as more open and extraverted than prior to the job change. In sum, employees who experience job changes thus encounter situations with a new trait-activation potential and by behaving according to the requirements of those situations, certain personality characteristics may be enhanced. Support for the relevance of situations’ trait-activation potential in the work context stems from a recent study by Judge and Zapata [58]. These authors found that the Big Five predicted job performance particularly well when the job context was relevant for respective personality characteristics. For example, openness to experience was a particularly strong predictor of job performance in situations requiring creativity, while extraversion played a key role in contexts involving social interactions.

Another theoretical underpinning for effects of job changes on changes in personality characteristics stems from social investment theory [57]. It argues that “investing in social institutions, such as age-graded social roles, is one of the driving mechanisms of personality development” ([54], p. 8). The theory purports that as individuals enter certain life roles, such as marriage or the workforce, they make a psychological commitment to those roles. In order to fulfill the social expectations associated with certain life roles, individuals’ personalities may shift accordingly. Applied to the context of this study, employees psychologically commit to and invest in their new roles as they enter managerial and professional positions. Since those positions are associated with certain behavioral expectations, such as being open to new experiences or extraverted, employees may behave accordingly. Their personalities subsequently shift according to those expectations. Supporting the theoretical propositions of social investment theory, Hudson, Roberts, and Lodi-Smith [59] found that social investment at the workplace indeed affected personality development. More particularly, results suggested that employees who cognitively and emotionally invested in their jobs showed both cross-sectional and longitudinal changes in their Big Five personality characteristics. Social investment theory further suggests that the reciprocal influences between personality characteristics and work experiences are likely to be corresponsive, as posited in the corresponsive principle [52]: the same characteristics that predict specific work experiences are the ones that are more likely to change due to those experiences. Based on trait activation theory, social investment theory, and the corresponsive principle, we thus propose that openness to experience and extraversion not only influence upward job changes into managerial and professional positions, but that such job changes also influence openness to experience and extraversion over time. For the other three characteristics of the Big Five framework, we again offer no directional hypotheses, but argue why we expect no reverse effects of those personality characteristics on upward job changes into managerial and professional positions.

#### Openness to Experience

According to trait-activation theory [56] and social investment theory [57], managerial and professional positions would have to confront employees with situations in which they are expected to behave openly in order to evoke changes in their openness to experience. Upward job changes into these positions indeed entail new situations that require employees to adapt to new people with ideas and opinions different from their own, new job requirements, and new environments [20]. The new work requirements of employees in managerial and professional positions furthermore call for creative solutions and divergent thinking [60], which are key aspects of openness to experience [34]. When taking on leadership roles, there may be especially many novel situations and unforeseen changes [61] with a high trait-activation potential for openness to experience, so that this personality characteristic may be enhanced due to upward job changes into managerial and professional positions. Research has shown that individuals with high openness to experience “are better able to understand and adapt to others’ perspectives” ([62], p. 754). When faced with the challenges of new jobs on higher hierarchical levels, these employees should be able to master them particularly well, which in turn is likely to positively impact on their openness to experience. Since we argue that openness to experience serves as a predictor of upward job changes into managerial and professional positions, the corresponsive principle [52] would suggest that employees also become more open in response to those job changes.

*Hypothesis 3*: Upward job changes into managerial and professional positions predict increases in openness to experience over time.

#### Extraversion

Extraversion is particularly relevant in social interactions and in leadership roles [4]. In response to upward job changes into managerial and professional positions, employees have to adapt to new social environments and interact with relevant others in order to build a professional network [63]. In addition, they may be required to take on a leadership role and exert social dominance in their new position [64]. According to trait-activation theory [56], the situations in managerial and professional positions should thus have a high trait-activation potential for extraversion, such that employees may become more extraverted in response to upward job changes into such positions. Social investment theory [57] would furthermore suggest that managerial and professional positions, which require networking and potentially leadership behaviors, are tied to expectations of being extraverted. Therefore, upward job changes into managerial and professional positions should have the potential of increasing employees’ extraversion. Since we argue that extraversion serves as a predictor of upward job changes into such positions, the corresponsive principle [52] would suggest that extraversion should also be enhanced in response to those job changes. Overall, we assume that extraversion increases in response to the exposure to and increased practice of dealing with social and leadership requirements that accompany upward job changes into managerial and professional positions.

*Hypothesis 4*: Upward job changes into managerial and professional positions predict increases in extraversion over time.

#### Conscientiousness

Employees who experience upward job changes into managerial and professional positions may increase in conscientiousness because they need to prove themselves in their new work environments. They may become especially dutiful and self-disciplined and try to avoid mistakes in order to make a good impression on their new superiors and colleagues. Also, conscientious employees may try to do their new managerial or professional position justice by working in an especially conscientious way. On the other hand, one may argue that when employees enter a new job, especially one in a higher hierarchical position, they may already have achieved their goal of being promoted. They may then have a lesser need for being conscientious and achievement-striving at work. Since there is reason to assume that upward job changes into managerial and professional positions may either enhance or limit employees’ conscientiousness, no directional hypothesis is offered here.

#### Agreeableness

Employees who experience upward job changes into managerial and professional positions need to adapt to new social structures in different work environments with new colleagues and superiors. Therefore, one could argue that their agreeableness may increase in response to those novel and diverse social interactions, particularly if those social interactions occur with decision-makers in the organization. On the other hand, employees who experience upward job changes, especially into leadership positions, may be required to behave in a less agreeable way in order to successfully fulfill their leadership tasks, for example when tasks have to be delegated to subordinates or in situations requiring negotiation skills. Thus, agreeableness could increase or decrease in response to upward job changes into managerial and professional positions.

#### Emotional Stability

Job changes, especially into higher hierarchical positions, typically involve tasks with more task-related and social responsibilities. Employees experiencing those upward changes may be insecure about their new duties and responsibilities, which may become evident in higher levels of neuroticism and thus lower levels of emotional stability. On the other hand, changing jobs may also increase individuals’ emotional stability, for example when they escape the undesirable circumstances of their previous job on lower hierarchical levels or when they regard becoming promoted into leadership positions as a consequence of their success at work. One could thus argue that emotional stability may either decrease or increase in response to upward job changes into managerial and professional positions.

## Methods

### Participants

To test our hypotheses, we used data from the Household, Income and Labor Dynamics in Australia (*HILDA*) survey, a national representative panel study that has been conducted annually since 2001 and surveys approximately 20,000 individuals each year [65]. All publications that have ever used the HILDA dataset can be obtained from the University of Melbourne Institute of Applied Economic and Social Research website [66]. To the best of our knowledge, no study has previously used the HILDA dataset to investigate relationships between personality characteristics and upward job changes into managerial and professional positions. Studies have, however, investigated the predictive role of personality characteristics in explaining occupational choice [67] and occupational change [68]. While Ham and colleagues [67] focused on personality as a predictor of belonging to white and blue color occupations, Carless and Arnup [68] made use of a question in the HILDA that asked respondents whether they occupation has changed since the last interview. Respondents were informed that “a promotion or a change in employer does not necessarily mean a change in occupation” (p. 85) and the authors point out that their operationalization of occupational change “is *not* a promotion or job change” (p. 85). Both studies thus investigated the effects of personality characteristics on occupational choices, however focusing on constructs that clearly differ from the one used in the present study, namely upward job changes into managerial and professional positions. Furthermore, both studies did not consider reverse effects of personality characteristics on job changes.

The longitudinal design of the HILDA survey enabled us to investigate the effects of the Big Five on subsequent job changes and the effects of job changes on changes in personality characteristics over time. It also allowed us to control for a number of potentially important confounding variables (i.e., age, gender, educational background, and tenure in the occupation) that may influence the relationships between the variables of interest. We only included participants for whom information on personality characteristics in the years 2005 and 2009 as well as job status for all of the measurement waves 2005 through 2009 was available, resulting in a sample of *N* = 3,489 participants. The mean age of the sample was 42.60 years (SD = 11.27), with almost even proportions of male and female participants (47% female, 53% male).

### Measures

We extracted variables measured between 2005 and 2009 from HILDA. We chose the year 2005 as starting point because the Big Five were measured for the first time in that wave. The Big Five were assessed for a second time in 2009. Data from the measurement waves 2005 through 2009 were used to operationalize job changes between the waves. The resulting data were therefore longitudinal and allowed investigating the effects of the Big Five assessed in 2005 on subsequent job changes between 2005 and 2009 as well as the effects of job changes on the Big Five assessed in 2009 (taking into account the Big Five measured in 2005).

### Big Five Characteristics

Openness to experience, extraversion, conscientiousness, agreeableness, and emotional stability were assessed in 2005 and 2009 with 28 items based on the well-validated Big Five mini-markers scales developed by Saucier [69]. Respondents were asked how well 28 adjectives describe them on a 7-point scale ranging from 1 (*does not describe me at all*) to 7 (*describes me very well*). We reversed items that were phrased negatively and calculated the scale means by dividing the sum of all item scores by the number of items for each of the Big Five personality characteristics. Internal consistency reliability estimates of all scales were satisfactory with all Cronbach’s *α*s ≥ .74 (also see Table 1). Principal components analyses with Varimax rotation furthermore revealed that the factor loading of the rotated solution showed that all items had their highest loading on their designated latent factor, with all factor loadings ≥ 53 (for a list of all items and their factor loadings, see S1 Table).

**Table 1:** Descriptive Statistics and Correlations of Variables.

Variable		*M*	*SD*	1	2	3	4	5	6	7	8	9	10	11	12	13	14	15
1	Age	42.60	11.27	-														
2	Gender	0.47	0.50	.07***	-													
3	Education	4.65	2.63	-.08***	-.02	-												
4	Tenure in occupation	9.59	9.74	.33***	-.07***	.10***	-											
5	Openness 2005	4.28	1.02	-.03	-.02	.22***	-.02	.74										
6	Extraversion 2005	4.44	1.08	-.04**	.14***	-.01	-.06***	.05**	.78									
7	Conscientiousness 2005	5.13	1.00	.06**	.12***	.09***	.08***	.04*	.12***	.79								
8	Agreeableness 2005	5.36	0.88	.04*	.29***	.03	.01	.25***	.15***	.26***	.81							
9	Emotional stability 2005	5.15	1.04	.10***	.03	.05**	.07***	-.17***	.19***	.27***	.16***	.75						
10	Upward job change	0.13	0.33	-.07**	.02	.23***	-.06**	.14***	.03	-.01	.05*	.02	-					
11	Openness 2009	4.22	1.02	-.02	-.04*	.22***	-.01	.74***	.05**	.03	.16***	-.08***	.15***	.75				
12	Extraversion 2009	4.42	1.08	-.03	.13***	-.02	-.05**	.04*	.78***	.09***	.13***	.14***	.02	.06**	.79			
13	Conscientiousness 2009	5.18	0.97	.05**	.14***	.05**	.06***	.02	.11***	.72***	.19***	.21***	.00	.06**	.11***	.80		
14	Agreeableness 2009	5.36	0.87	.06**	.29***	.03	.03	.17***	.16***	.19***	.66***	.17***	.03	.26***	.17***	.26***	.80	
15	Emotional stability 2009	5.25	1.01	.10***	.05**	.05**	.07***	-.08***	.15***	.23***	.19***	.66***	.02	-.15***	.18***	.28***	.19***	.81

*N* = 3,489. Cronbach’s alphas, where available, are shown along the diagonal.

* *p* < .05.

** *p* < .01.

*** *p* < .001.

#### Upward Job Changes into Managerial and Professional Positions

Upward job changes into managerial and professional positions were operationalized on the basis of the coding scheme provided by the Australian and New Zealand Standard Classification of Occupations (*ANZSCO*). The ANZSCO is a skill-based classification system that aims to catalogue all occupations in the Australian labor market [70]. It makes use of eight major groups (managers, professionals, technicians and trade workers, community and personal service workers, clerical and administrative workers, sales workers, machinery operators and drivers, and laborers), all of which are again divided into several sub-major groups (such as education professionals, health professionals, etc.).

In the ANZSCO, each of the sub-major groups is assigned a particular skill level that is required for working in that occupation. Skill levels range from 1 to 5 and are defined by the range and complexity of the tasks that are performed in an occupation and are operationalized as the level and amount of formal education and training, previous experience, and on-the-job training required for working in the occupation. Thus, the coding of skill levels in ANZSCO is very similar to the ‘job zones’ used in the O*NET in the United States ([71], see also http://www.onetonline.org/find/zone). In the major groups of managers and professionals, almost all of the occupations (except hospitality, service, and retail managers and farmers, all of which were therefore not included in the sample) are assigned the highest possible skill level (i.e., 1), while none of the other major groups are assigned the highest skill level. Therefore, working in managerial and professional positions requires a higher set of skills than any other position in ANZSCO and takes place in hierarchically higher positions. We would like to point out that this operationalization does not distinguish between within- and between-organization job changes, but solely focusses on the above-mentioned changes in skill levels associated with the positions that individuals work in.

In the HILDA survey, participants are asked to describe the occupation and industry of their current job, and their verbatim responses are immediately coded by the HILDA interviewer. HILDA does not report evidence of the reliability and validity of this procedure. However, the coding is very similar to the procedure used by O*NET to code job zones, which has been found to be reliable and valid with inter-rater reliabilities of at least .70 [71]. In the present study, participants in the full sample of *N* = 3,489 individuals were coded as having made an upward job change into managerial and professional positions (i.e., a score of 1) if they had changed their occupation from non-managerial and non-professional positions to managerial and professional positions and remained in such positions in the timeframe of 2005 through 2009. If they remained in non-managerial and non-professional positions associated with a lower skill-level within the same timeframe, this was coded as no upward job change into managerial and professional positions (i.e., a score of 0). This procedure resulted in a sub-sample of *N* = 247 participants who experienced an upward job change into managerial and professional positions and *N* = 1,710 participants who remained in non-managerial and non-professional positions.

#### Control Variables

In all analyses, we controlled for age, gender, educational level, and tenure in the occupation. Age was included as a control variable because personality changes across the lifespan [50]. Gender was controlled in the analyses because careers of men and women develop differently [72]. Finally, educational level and tenure are two main predictors of job attainment [73]. Educational level was measured on a 9-point scale ranging from 1 (*year 11 in high-school or below*) to 9 (*master or doctoral degree*). Tenure in the occupation was operationalized as the number of years the participant has worked in the same occupation prior to the change reported in the timeframe of the study.

### Statistical Analyses

Since data was missing completely at random, we used maximum likelihood estimation to impute missing data in the control variables [74], which was present for less than 1% of cases. Subsequently, we used two different methodological approaches to test our hypotheses.

First, we employed event history analyses using the SPSS software, also known as survival analyses [75], to estimate the effects of the Big Five on upward job changes into professional and managerial positions. Event history analysis not only estimates whether an event occurred or not, but also takes into consideration the time it took for the event to occur. This analytical approach thus treats job change as a time-dependent variable rather than a binary variable. Furthermore, event history analysis accounts for censored data. The observation period of the present study ended after 2009, but it is possible that individuals experienced upward job changes into managerial and professional positions after that. Our data were therefore right-censored, and event history models accounted for this. In the event history analyses, we entered all control variables in a first step of the Cox regression hazard rate models [76], the personality characteristics measured in 2005 in a second step, and the time until the upward job changes occurred as the time variable. Note that this statistical approach controls for correlations between the personality factors—that is, each independent variable’s effect is independent of the other effects.

Second, we employed propensity score matching ([77], for a recent overview, see [78]) for testing the hypotheses concerned with the effects of upward job changes into managerial and professional positions on changes in personality. Researchers have suggested that propensity score matching is the method of choice when estimating causal effects of group membership on the basis of observational data [79]. When participants cannot be randomly assigned to experimental conditions, such as in our study to upward job change into managerial and professional positions versus no change into such positions, a comparison between those experimental conditions may be distorted [80]. Propensity score matching aims at reducing this bias by pairing participants from the different experimental conditions who are similar in terms of certain pre-defined covariates. We included the control variables (age, gender, education, tenure in the occupation) as well as the Big Five measured in 2005 into the model as covariates, since pretest scores are especially important covariates [81].

Using the MatchIt software package for SPSS [82], we estimated a propensity score for each participant, which is a measure of the likelihood of a person’s group membership given the observed covariates. Participants from both groups were then matched using a 1:2 nearest neighbor matching. Consistent with previous research, we imposed a caliper of .20 of the standard deviation of the propensity score’s logit to avoid matches of very diverging propensity scores [51]. After the matching, none of the standardized mean differences between the covariates were above d = .20, so that we could conclude that the matching improved the overall balance between the groups. The matching procedure resulted in a sample of *N* = 687 participants for these analyses. Based on this matched sample, we then conducted group comparisons (independent sample t-tests) for estimating whether individuals who experienced an upward job change into managerial and professional positions differed from participants who did not experience such a change in terms of their subsequent scores on the Big Five assessed in 2009.

## Results

Table 1 presents the means, standard deviations, and correlations of variables.

### Effects of the Big Five on Upward Job Changes into Managerial and Professional Positions

As outlined in the Method section, we used Cox regression hazard rate models to assess the effects of the Big Five on upward job changes into managerial and professional positions. Results can be found in Table 2 and indicate that openness to experience significantly and positively predicted upward job changes into managerial and professional positions (*Β* = .33, *p* < .001). The effect size suggested that a one-unit increase in openness to experience was associated with a 39% higher likelihood of experiencing upward job changes into managerial and professional positions. None of the other Big Five characteristics had a statistically significant effect on upward job changes into managerial and professional positions (see Table 2). Results thus offered support for Hypothesis 1, but not for Hypothesis 2, suggesting that openness to experience—but not extraversion and the other Big Five characteristics—had an effect on upward job changes into managerial and professional positions.

**Table 2:** Results of a Cox Regression Hazard Rate Model Predicting Upward Job Changes into Managerial and Professional Positions.

	Step 1 (control variables)			Step 2 (personality characteristics)		
Predictor variables	*B*	*SE*	*OR*	*B*	*SE*	*OR*
Age	-.01	.01	.99	-.01	.01	.99
Gender	.15	.13	1.17	.17	.14	1.18
Education	.29***	.03	1.34	.27***	.03	1.31
Tenure in Occupation	-.02*	.01	.98	-.02*	.01	.98
Openness 2005				.33***	.07	1.39
Extraversion 2005				.05	.06	1.05
Conscientiousness 2005				-.12	.07	.88
Agreeableness 2005				.01	.09	1.01
Emotional stability 2005				.11	.07	1.12

*N* = 1,957. SE = Standard errors. OR = odds ratio.

* *p* < .05.

*** *p* < .001.

### Effects of Upward Job Changes into Managerial and Professional Positions on Changes in the Big Five

**Fig 1:**
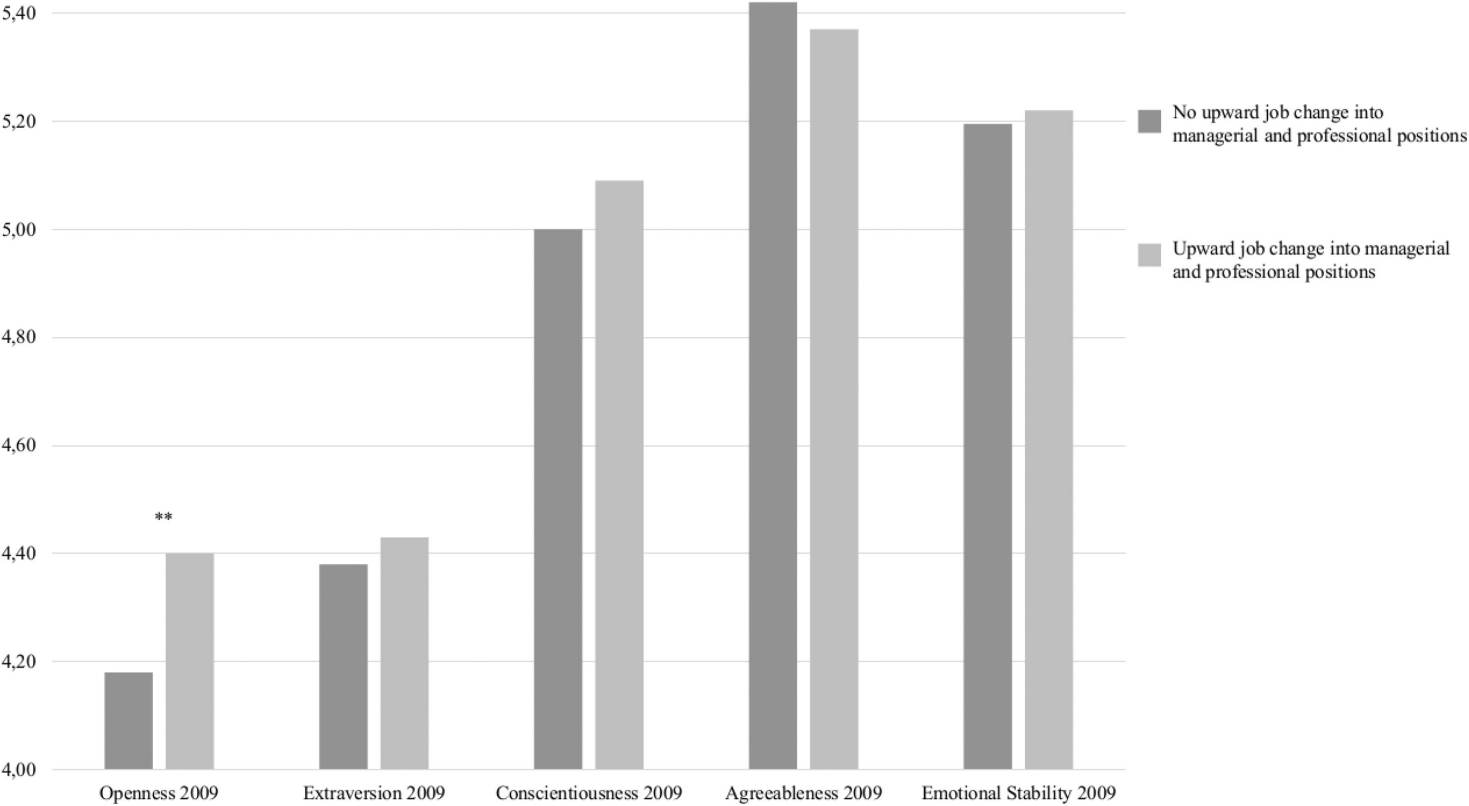
Means of personality characteristics in 2009 for participants who experienced no upward job change into managerial and professional positions and participants who experienced an upward job change into managerial and professional positions.

For estimating the effects of upward job changes into managerial and professional positions on changes in the Big Five, we made use of group comparisons on the basis of the matched sample that had resulted from the propensity score matching procedure outlined in the Method section. This procedure ensured that control variables and initial levels of personality characteristics were accounted for. Results (see Fig 1) indicated that participants who experienced upward job changes into managerial and professional positions were significantly higher in subsequent openness to experience (*M* = 4.40, *SD* = .06) than participants who did not experienced such changes (*M* = 4.18, *SD* = .05; *t*(685) = 2.81, *p* = .005). This difference in means corresponds to an effect size of Cohen’s *d* = .21, which would be considered a small effect [83]. Fig 1 further shows that individuals who experienced upward job changes into managerial and professional positions did not differ significantly from individuals who did not experience such job changes in terms of extraversion and any of the other Big Five characteristics. Results therefore offered support for Hypothesis 3, but not for Hypothesis 4.

## Discussion

Over the past decade, theory and empirical research have accumulated suggesting that personality characteristics do not only predict work experiences, but that work experiences also may lead to changes in personality characteristics over time [11, 16, 52]. The overarching goal of this study was to test this notion of reciprocal effects between personality and work with regard to upward job changes into managerial and professional positions, which represent important career transitions [20] and are relevant indicators of employees’ career success [7].

We first examined effects of the Big Five on upward job changes into managerial and professional positions. We therewith extended existing empirical findings supporting the theory of vocational choice [26], person-environment fit theory [27, 28], and the attraction-selection-attrition model [29] by investigating whether personality characteristics not only predict initial job choices, but also changes in individuals’ careers. Our results indicated that openness to experience played a key role in explaining upward job changes into managerial and professional positions, while the remaining four personality characteristics in the Big Five framework had no statistically significant effects. Employees who are particularly open to experience seem to either self-select or be promoted into managerial and professional positions. This may be due to the fact that openness to experience is associated with intellectual ability and flexibility, divergent thinking, and the generation of new ideas, all of which seem to be especially important in managerial and professional positions.

Extraversion, which we also expected to predict such upward job changes, did not emerge as a statistically significant predictor of upward job changes into managerial and professional positions. Based on several of the facets of extraversion, such as ambition, assertiveness, and social dominance, one would have expected this personality characteristic to play a role in predicting job changes into hierarchically higher positions. However, extraversion is also associated with high levels of career and job satisfaction, which may lead extraverted employees to not actively initiate upward job changes. Even employees who are selected by higher management to shift to a higher position would also have to make their own decision to accept such a position. If they were entirely satisfied with their current situation, they would have the opportunity of rejecting such an offer from higher management. Our finding from an Australian sample complements previous research, which has shown that extraversion serves as a predictor of objective career success in Europe, but not in the United States [47]. Since Australian culture is similar to the United States culture [84], scoring relatively high on individualism and indulgence and relatively low on long-term orientation and power distance, this finding does not come as a surprise. While the importance of openness to experience for upward job changes is consistent with theoretical accounts of the role of personality characteristics for job changes [20, 42], the same does not hold for extraversion. Future research should aim at examining potential explanations for this unexpected finding.

Second, we investigated whether upward job changes into managerial and professional positions also lead to changes in personality characteristics over time based on the guiding framework of the DDM of personality and work [16]. Our results suggested that upward job changes into professional and managerial positions predicted only increased levels of openness to experience but not the other Big Five characteristics. When employees are confronted with novel situations and role expectations in managerial and professional positions, their level of openness to experience seems to shift accordingly. This finding may be explained by the notion that employees in managerial and professional positions may frequently encounter challenging situations that require them to make use of their divergent thinking skills, their potential of generating new ideas, or their creativity, all of which are facets of openness to experience. Unexpectedly, we did not find an effect of upward job changes into managerial and professional positions on extraversion. This finding may suggest that the situations that employees encounter in such positions do not have a high trait-activation potential for extraversion. Moreover, extraversion may not be activated to the same extent in all types of managerial or professional positions. For example, a sales manager is more likely to encounter situations with a high trait-activation potential for extraversion than a research director. While the HILDA study does not provide specific information on the job titles or job descriptions of employees, we note that it involves a representative sample of employees and thus it is very likely that employees with a broad range of job titles and job descriptions were represented in our sample. Alternatively, the social expectations associated with managerial and professional positions may not be relevant for extraversion. For example, employees in managerial and professional positions may actually not be expected to exert social dominance [39], particularly shortly after entering such positions, so that behaving socially dominant would not be in line with expectations associated with the new role. Again, future research is needed to investigate why upward job changes into managerial and professional positions do not seem to play a role in shaping employees’ extraversion.

### Theoretical Implications

Overall, our findings offer some support for the core proposition of the DDM, which suggests that personality characteristics, such as openness to experience in our case, may not only predict relevant work experiences, but that work experiences can also lead to changes in relevant personality characteristics over time. Trait-activation theory [56] and social investment theory [57] provide two different theoretical bases to explain those results. When individuals change into higher hierarchical positions, they are confronted with situations that have a novel trait-activation potential compared to previous work-related situations. Through upward job changes into managerial and professional positions, employees furthermore enter and commit to new social roles that are associated with specific social expectations. Due to the opportunity to behave according to the trait-activation potential of the newly encountered situations and in order to fulfill the associated social expectations, personality characteristics relevant to the new job can become more pronounced. This finding extends previous research, which has shown that work characteristics can influence employees’ daily manifestations of personality [85], by showing that upward job changes can likewise have an effect of changes in personality characteristics. In the context of the present study, this means that employees become more open to experience in response to upward job changes into managerial and professional positions. In the present study however we do not differentiate between the two different potential explanatory mechanisms posited in trait-activation theory [56] on the one hand and social investment theory [57] on the other. Further research is needed to better understand the mechanisms through which work-related experiences can lead to changes in personality characteristics.

Moreover, the results of our study support the corresponsive principle in the personality literature [52], suggesting that openness to experience predicts upward job changes, and that the same personality characteristic is enhanced by these work-related experiences. The corresponsive principle may be particularly relevant for cross-sectional research findings that have established a relation between personality characteristics and work outcomes. These findings may well have supported the predictive validity of personality in industrial and organizational psychology, but may have missed that work experience can also shape the same personality characteristics that have led to the work experience in the first place. Future research could thus greatly benefit from reexamining well-established relationships between personality and work outcomes by also investigating whether reverse effects of personality characteristics on work outcomes exist. Such research would furthermore be able to detect potential ceiling effects that could occur if already distinct personality characteristics keep being enhanced by work experiences over time.

The findings of the present study also suggest that upward job changes into managerial and professional positions only shape a single personality trait, namely openness to experience, and that the effect of job changes on openness is rather small. One might even question whether the measured change in openness to experience indeed reflects a change in personality or rather a change in personality-related behavior. We argue based on the DDM that work-related changes first lead to changes in personality-related behaviors, which then stabilize over time to become part of people’s stable personality tendencies. Furthermore, these behaviors are likely to be maintained over time, for instance because people are rewarded in work settings for behaving according to the expected norms. Future research is needed to investigate whether the effect of upward job changes on openness to experience is persistent over time and across different operationalizations of the constructs.

Picking up on the long-running person-situation debate in psychology, Judge and Zapata [58] have recently shown that employees are particularly likely to express certain personality characteristics when the situations they find themselves in activate those characteristics. Applied to the context of the present study, this finding could have two implications. First, work experiences may have to be relevant for certain personality characteristics in order for those characteristics to be enhanced over time. This could be an explanation for the finding that upward job changes into managerial and professional positions only had an effect on employees’ openness to experience, but not on any other Big Five characteristic. Second, situational cues may have to be even stronger in order to produce more pronounced changes in personality characteristics. However, job changes into managerial and professional positions constitute already quite strong situational influences that occur relatively rarely in work settings, suggesting that the practical usefulness of the relatively small effect found in this study may be limited. However, we argue that the effect of job changes on openness found in this study over a 5-year period is meaningful given that personality has been conceived as a rather stable construct. Moreover, our operationalization of the Big Five did not measure the facet level of each personality construct. The effect of job changes on certain facets of openness (e.g., openness to new ideas, values, and actions) may be significantly stronger than the effect on the broader Big Five personality trait.

### Limitations and Avenues for Future Research

The present study has some limitations, which reveal promising avenues for future research. First, we cannot draw definite causal inferences based on our data, which was collected longitudinally, but was not based on an experimental research design with random assignment of participants to an experimental condition (upward job changes into managerial and professional positions) and a control condition (no upward job changes into managerial and professional positions). Such an ideal experimental design would be very difficult and unethical to implement in this area of research [86]. We however used a state-of-the-art methodological approach, propensity score matching, and combined it with longitudinal data collected over five years, which allows for more confident conclusions with regard to causality than traditional approaches [77, 78]. In this regard, our study may serve as an example for future studies that aim to examine the effects of naturally occurring group memberships on personal development as well as work and career outcomes.

Second, the time span of five years between the first and the last measurement wave used in the present study was arbitrary and lacks a solid theoretical justification [87]. To date, a theoretically driven estimation of what time span should be used when investigating reciprocal influences between personality and work experiences does not exist [16, 88]. Increased theorizing on the role of time for reciprocal relationships between personality and work experiences is thus needed. Future research could vary the time span under investigation and especially focus on the question of whether the reciprocal relationships between personality and job changes reported in this paper are sustained over time and additional measurement waves.

Third, the conceptualization and operationalization of both personality characteristics and upward job changes into managerial and professional positions used in this study may be criticized. The measurement of personality characteristics relied on Saucier’s mini-markers [69], which is a reliable and well-validated measurement instrument, but does not allow distinguishing between different facets of broader personality characteristics. Future research would benefit from using a more fine-grained measure of personality, such as the NEO-PI [31], to investigate how various personality facets influence upward job changes and vice versa. Due to our focus on such upward job changes, we furthermore did not provide any information on the reciprocal influences between personality and other forms of job mobility. Researchers have classified different types of job mobility into job changes, organizational changes, and occupational changes [20]. Thus, our operationalization may miss other important aspects of career-related changes. With our current data, we were also not able to investigate lateral job changes, such as taking on a similar job at a different organization, and we were not able to distinguish between voluntary and involuntary changes and between intra-organizational and inter-organizational changes. Another limitation is that no information on the validity and reliability of coding respondents’ verbatim responses into occupational categories is available in the HILDA survey. Future research could take a more fine-grained approach to allow more precise conclusions concerning the reciprocal relationships between personality and these types of career-related changes.

Fourth, our study does not provide insights into the mechanisms through which personality characteristics impact upward job changes into managerial and professional positions and vice versa. For example, the effect of openness to experience on upward job changes into managerial and professional positions may be driven by the fact that open individuals initiate certain occupational changes based on their disposition, or organizational decision makers may regard them as especially well-suited for creative tasks and select them based on those changes. The effect of upward job changes into managerial and professional positions on openness to experience could also be driven by different factors, such as using a larger variety of skills on the job, training opportunities, exposure to organizational decision-makers, leadership tasks, and international job experiences. Future studies should aim at identifying potential mechanisms that may explain the reverse effects of personality characteristics on upward job changes into managerial and professional positions.

## Conclusion

In the present study, we investigated reciprocal relationships between the Big Five personality characteristics and upward job changes into managerial and professional positions. Using a large longitudinal dataset, we showed that employees’ openness to experience not only predicted such job changes, but that the experiences made in managerial and professional positions also led to changes in this personality characteristic over time. These findings contribute to an emerging area in the literatures on career development and personality development by offering a dynamic perspective on the role of personality in the context of work and careers.

## Supporting Information

S1 Table Results of the Principal Component Analysis. (DOCX)
