# Supplementary material for: Openness to Experience as a Predictor and Outcome of Upward Job Changes into Managerial and Professional Positions
Source: PLoS One. 2015 Jun 25;10(6):e0131115. doi: 10.1371/journal.pone.0131115 (PMC4482250; doi:10.1371/journal.pone.0131115)
Supplement: S1 Table — (DOCX) [file pone.0131115.s001.docx]

**S1 Table**

**Results of the Principal Component Analysis.**

|  | A  2005 | C  2005 | O  2005 | ES  2005 | E  2005 | A  2009 | C  2009 | O  2009 | ES  2009 | E  2009 |
| --- | --- | --- | --- | --- | --- | --- | --- | --- | --- | --- |
| Sympathetic | .623 |  |  |  |  | .744 |  |  |  |  |
| Kind | .776 |  |  |  |  | .601 |  |  |  |  |
| Cooperative | .593 |  |  |  |  | .793 |  |  |  |  |
| Warmhearted | .731 |  |  |  |  | .641 |  |  |  |  |
| Orderly |  | .637 |  |  |  |  | .678 |  |  |  |
| Systematic |  | .394 |  |  |  |  | .728 |  |  |  |
| Inefficient (R) |  | .656 |  |  |  |  | .633 |  |  |  |
| Soppy (R) |  | .626 |  |  |  |  | .647 |  |  |  |
| Disorganized (R) |  | .724 |  |  |  |  | .391 |  |  |  |
| Efficient |  | .664 |  |  |  |  | .648 |  |  |  |
| Deep |  |  | .415 |  |  |  |  | .893 |  |  |
| Philosophical |  |  | .467 |  |  |  |  | .356 |  |  |
| Creative |  |  | .893 |  |  |  |  | .472 |  |  |
| Intellectual |  |  | .477 |  |  |  |  | .921 |  |  |
| Complex |  |  | .354 |  |  |  |  | .465 |  |  |
| Imaginative |  |  | .884 |  |  |  |  | .417 |  |  |
| Envious (R) |  |  |  | .534 |  |  |  |  | .554 |  |
| Moody (R) |  |  |  | .713 |  |  |  |  | .726 |  |
| Touchy (R) |  |  |  | .594 |  |  |  |  | .596 |  |
| Jealous (R) |  |  |  | .582 |  |  |  |  | .581 |  |
| Temperamental (R) |  |  |  | .720 |  |  |  |  | .721 |  |
| Fretful (R) |  |  |  | .555 |  |  |  |  | .540 |  |
| Talkative |  |  |  |  | .557 |  |  |  |  | .441 |
| Bashful (R) |  |  |  |  | .558 |  |  |  |  | .460 |
| Quiet (R) |  |  |  |  | .683 |  |  |  |  | .765 |
| Shy (R) |  |  |  |  | .762 |  |  |  |  | .692 |
| Lively |  |  |  |  | .462 |  |  |  |  | .556 |
| Extroverted |  |  |  |  | .435 |  |  |  |  | .553 |

A = Agreeableness; C = Conscientiousness; O = Openness; ES = Emotional Stability; E = Extraversion; (R) = reverse coded.
